# Emerging Technologies, STI Diaspora and Science Diplomacy in India: Towards a New Approach

**DOI:** 10.3389/frma.2022.904100

**Published:** 2022-06-22

**Authors:** Nimita Pandey, Krishna Ravi Srinivas, T. R. Deepthi

**Affiliations:** ^1^DST - Centre for Policy Research, Indian Institute of Science, Bangalore, India; ^2^Research and Information System for Developing Countries, New Delhi, India; ^3^Centre for Canadian, US and Latin American Studies, Jawaharlal Nehru University, New Delhi, India

**Keywords:** Science Diplomacy, emerging technologies, SDGs, diaspora, brain gain

## Abstract

Utilizing the expertise and knowledge resources of the diaspora, particularly the scientific diaspora, has been part of the strategies of many countries. In the recent years, realizing the importance of the potential of the diaspora to contribute to national development and Science, Technology, and Innovation ecosystem, countries have used Science Diplomacy also to engage with the scientific diaspora. Science Diplomacy is hailed as an enabler and facilitator and is often seen in the context of international S&T collaboration or big science projects. But the use of Science Diplomacy for diaspora engagement calls for specific strategies and meaningful initiatives. India is one of the major developing countries that has given a major thrust to engaging with the scientific diaspora. India is also a leading player in the global Science Diplomacy arena. This article critically examines India's initiatives and strategies for engagement with the scientific diaspora. It points out that the Science Diplomacy dimension is missing in this. Using examples from other countries, recent thinking, and developments in Science Diplomacy, this study outlines an approach with some examples of strategies and initiatives for harnessing Science Diplomacy to enhance engagement with the scientific diaspora and create a win-win milieu for India and the diaspora. The approach takes into account the proposed and ongoing initiatives in emerging technologies in India, including quantum technologies and Artificial Intelligence. Such a framework will create a synergy among various programs and initiatives by using Science Diplomacy as a facilitator and catalyst. Under this framework, Diaspora is involved not only as experts and contributors to scientific advancements but also as stakeholders. This dual role of the STI Diaspora can bring a paradigm shift in traditional understanding and use of science diplomacy, particularly to engage and harness the potential of the STI Diaspora for Sustainable Development.

## Introduction

At a juncture where non-state actors are gaining prominence in building bonds between nation-states, science diplomacy becomes an avenue worth exploring in policymaking. Science benefits the entire humanity and transcends boundaries of language, ethnicity, and race, particularly in its ability to make human life easier. From the perspective of developing countries, this makes even more sense as the access to technology and knowledge from more advanced countries can be a great help. However, various barriers in the form of economic and narrow short-sighted political interests come in the way (Gottstein, 2003). This is where engagement with the diaspora of scientists, engineers, and doctors becomes effective.

Highly educated professionals command respect irrespective of how their country of origin is perceived in the host nation. Gottstein (2003) cites the instance of American culture being negatively perceived in Islamic countries and yet the scientific and technological achievements of that country being highly respected and envied. This shows the potential of science to build bridges where differences in culture and politics may be divisive. For countries from the Global South, diaspora presents untapped potential in this regard.

The discussions on “Brain-Drain” and “Brain-Gain” to “Brain-Circulation” and beyond signify the importance of diaspora. Discussion on brain-drain started in the 1960s and at that time the connotation was by and large negative. For example, according to UNESCO (Siegfried and Singh, 1987), “the brain-drain could be defined as an abnormal form of scientific exchange between countries, characterized by a one-way flow in favor of the most highly developed countries” (Siegfried and Singh, 1987). In this article, we adopt the definition of diaspora “as a category of practice, project, claim, and stance, rather than as a bounded group” (Brubaker, 2006). This definition, while going beyond the traditional usage of the term, facilitates conceptualizing diaspora in a broader sense, including practices, networks, mobilizations, and projects/programs.

Because of various factors, the migration of students, scientists, academics, and entrepreneurs has been continuing, and even when countries are offering more opportunities to the scientists and technocrats, they are also aware that migration need not be looked at as a purely negative phenomenon.

The report of the Global Commission on International Migration stated that Country of Origins (COs) should “establish an inventory of the skills base within the diaspora; develop programs that facilitate the transfer of skills and knowledge from the diaspora to their COs” (Global Commission on International Migration, 2005). For countries like Brazil, the Philippines, China as well as European countries like Greece and Ireland, harnessing the skills and talents of the diaspora, leveraging their networks, and connections for national development has been a priority, which is very well-manifested by them adopting different strategies. For example, Brazil adopted Rede Diáspora Brasil, as a plan to engage with the Brazilian diaspora of STI (Maastricht Centre for Citizenship Migration Development, 2021). Interestingly, it has also been suggested that ASEAN countries can complement their efforts by establishing a collaborative platform to pool the expertise as well as the transnational national networks of their Highly Skilled Diasporas (HSD) (Fok et al., 2021).

But among the diaspora, the STEM and/or STI diaspora, which consist of scientists, and technocrats, has a unique place on account of the importance of STI for national development and the central role of STI in economic progress. Hence, many countries including India are making efforts to incentivize these communities for contributing to capacity building in STI.

On the other hand, brain circulation and migration of talented persons including experts is likely to continue on account of the following factors which will result in the growth and expansion of the professional diaspora: (1) Globalization of the economy, STI, knowledge, and greater integration of countries with global systems; (2) Countries (e.g., Singapore) wooing talented persons from other countries to meet their needs and to expand their STI infrastructure, by offering incentives, granting citizenship on easier terms, and promoting expertise and entrepreneurship in selected sectors; (3) Young professionals and students with aspirations to move abroad for education and career; (4) the strengthening of ties of diasporas with their homelands, incentivizing migration as an option and as a normal phenomenon; and, (5) increase in international engagement of firms from the Global South, necessitating migration of talented personnel.

Moreover, the next generations among diasporas are likely to have ties with the homelands of the previous generations, irrespective of how strong, or weak the ties are. So, a pragmatic approach will be required to think in terms of “brain gain”, “brain circulation” and making the best use of professional diaspora knowledge, expertise, and connections than to bemoan migration or reluctance of students to return after completing education/training.

In this context, more than one strategy and a multi-pronged approach are needed, including leveraging soft power. But as nations pursue talented human resources in STEM and make that part of their innovation strategy, it is not easy for developing countries to make the most of brain circulation. For example, the United Kingdom's innovation strategy states “Priority 1.1: Make the United Kingdom the most attractive destination for talented people and teams from the United Kingdom and around the world” (United Kingdom Research and Innovation, 2022). While, this dovetails with the United Kingdom's ambitions in Science, Technology, and Innovation, such strategies may also induce the migration of talented human resources afresh.

In the case of India, while there has been “brain-drain”, diffusion of knowledge through migration of scientific labor is also important and there are opportunities and constraints in making use of diffusion of their knowledge (Kale et al., 2008). India has two categories of diaspora, i.e., Non-Resident Indians and Overseas Citizens of India (OCI). Overseas Citizenship of India (OCI) was introduced as a strategy for strengthening the ties of this diaspora with India so that dual-citizenship will be a win-win for them and India.

Recent research has thrown light on how to make the process of migration beneficial for both countries of origin and the destination; one of the suggestions offered is a pre-migration agreement for jointly financing the education and training of the migrants (Clemens, 2015). This might prove to be difficult since individuals can always have a change of plans regarding migration and when exactly to handpick migrants or provide them with the option to opt in, all pose tricky questions. Initiatives to ensure greater engagement targeted at scientists specifically, done with clear objectives in mind and implemented in a multidimensional manner, can reap great benefits.

The STEM diaspora, while contributing to global S & T, also contributes to the growth and development of STI in their homelands, particularly in new and emerging technologies. A study of the Chinese Diaspora and their contributions show that the Chinese diaspora contributed to the Chinese catching up in global science and science during 2000–2015 (Xie and Freeman, 2020). In the 1980s, Ireland formulated a series of policies for mobilizing diaspora contributions to Ireland in multiple ways resulting in inter alia, a larger inflow of FDI, and the promotion of tourism (Tian and Wu, 2016). Indian studies have established the positive contributions of diaspora and this could result in a win-win for the country of origin and country of destination (Buga and Meyer, 2012).

Apart from direct financial and intellectual contributions to developmental projects, their outreach to the citizens back home could provide valuable information on opportunities and access to their professional networks (Meyer and Brown, 1999); this can be utilized by NGOs and startups. Young minds can be broadened with the right kind of exposure and the inculcation of a scientific temper. The sharing of their experience would enable us to make desirable policy changes, particularly in our academic institutions so that we can provide the kind of institutional support the diaspora enjoys in more technologically advanced countries. This has been captured as “informational”, “reputational”, and “cultural” diffusion resulting from diaspora engagement (Paul, 2012).

Science diplomacy also becomes an opportunity for countries like India to take on the leadership in issues that really matter and thereby gain more respect and appreciation in the international community, particularly at a time when the superpowers have been hesitant to assume similar roles. At a regional level, it can provide a more compassionate leadership due to the understanding of Global South perspectives that it shares with its neighbors. India has already established the International Solar Alliance aimed at promoting solar energy worldwide and done its share in making COVID-19 vaccines available to the rest of the world. The expertise and networks of Indian-origin scientists can contribute greatly to such endeavors as well.

Over the years, the Government of India has initiated specific programs on using the expertise and skills of the STEM diaspora and engaging with them for mutual benefit (for details, see Initiatives in India section). This is done with an understanding that while they would work with institutions and initiatives, it would be a win-win situation. As we discuss elsewhere, these programs have been useful but not sufficient in terms of numbers or scope to make the best use.

Like many other governments, the Government of India has been enhancing its efforts for greater and better engagement and the recent PRABHASS is one such initiative (more details on https://www.prabhass.gov.in/). However, such efforts are to be seen in the context of Science Diplomacy (SD) to make the engagements more useful, dynamic, and meaningful. The section “Toward a New Model for Development: Linking Science Diplomacy and STI Diaspora” of the article proposes some approaches in this regard.

## Approaches and Strategies in Using Diasporas and Networks

“Diaspora networks—of Huguenots, Scots, Jews, and many others—have always been a potent economic force, but the cheapness and ease of modern travel have made them larger and more numerous than ever before. There are now 215 million first-generation migrants around the world: that's 3% of the world's population. If they were a nation, it would be a little larger than Brazil” (The Economist, 2011).

“Brain-drain” from India has attracted the attention of the government and other stakeholders since the 1970s. While, the negative connotation has, by and large, vanished, it is still a matter of concern, particularly the migration of students for education. According to the Organization for Economic Co-operation and Development (OECD), India has 3.12 million highly educated migrants. Although the number of Indians who gave up Indian citizenship has been increasing, the official contention was “no significant brain-drain to such an extent of affecting the developments in the science and technology sector” (Mohan, 2022). In the literature, we find many analyses that trace the causes of the “brain-drain” and its scope and how this could be translated into “brain gain” (Lavakare, 2013). But it is also contended that while considering the huge population migration is not a significant one and India has one of the lowest migration rates in the world (Sharma, 2021).

Irrespective of the number of migrants or the size of the Indian diaspora what matters is their contribution and how this contribution is changing in terms of diversity and sectors. For example, diasporas and diaspora networks are playing a key role in startups in India in more than one way (Varma, 2020). Indian diaspora all over the globe have formed many kinds of networks and thanks to the information revolution these have increased and expanded, and they serve more than one function.

Initiatives like TIE based in Silicon Valley have played a major role in the transfer of technology to the Information Technology Enabled Services (ITES) sector in India and through chapters have assisted young technologists in managing the hi-tech business (Raj, 2012). But these days, governments and states encourage diasporas to get engaged with the state/country and help in the transformation of the economy and transition to the knowledge economy. For example, a recent initiative in the Kerala state in India is broad viz. “The scheme seeks to identify experts in high-tech industries dealing with intangible assets and invite them for redesigning the higher education curricula and reorienting the research domain” (George, 2021). Kerala is also building a database on its diaspora and their expertise.

While, it is obvious that diaspora can contribute in many ways, it cannot be assumed that it will happen on its own; well-defined and consistent policies and programs are needed (Siar, 2013). But neither diasporas nor their networks can be assumed to be free-floating, which could be tapped at one's own will. In fact, as Craven (2021) points out “In conclusion, whilst social connectivity is important, networks of diaspora engagement do not float freely. They are embedded in global and local cultural fields, which in turn are embedded in a material environment”.

STI policies are also paying attention to harnessing diasporas and their networks and want to integrate their plans for diaspora with overall STI policies trying to achieve more than one objective. and also link SD with this. For example, according to South Africa's white paper on Science, Technology and Innovation, “Properly leveraged, these ‘brain circulation' networks could help drive innovation and knowledge generation in South Africa, and improve science diplomacy between countries” (Department of Science and Technology Government of South Africa, 2019).

However, the challenges are many including calibrating effective programs, identifying the right kind of networks to engage with and linking SD with these. In the literature on engaging with the STEM diaspora, the potential of SD is barely acknowledged and vice versa. Moreover, there is not much literature on linking SD, STEM diaspora, and SDGs or emerging technologies. In this article, we address the three pointing out that India can and should leverage SD and STEM diaspora for achieving SDGs and use them in harnessing emerging technologies. We suggest that to achieve this, India needs a new approach and strategy that goes beyond traditional approaches in engaging with the STEM diaspora or using SD.

### Global Experience and Indian Experience in Engaging With STEM Diaspora and Science Diplomacy

Science diplomacy involving diaspora can go beyond merely reaching out to them or creating a common platform. It becomes imperative to look at the diaspora-oriented initiatives of other countries, particularly of those with similar socioeconomic conditions to improve our own. As UNESCO Science Report (2021) points out many countries have initiated different types of programs for engaging with the STEM Diaspora. While their scope varies, most of them are part of national STI policies/strategies (UNESCO Science Report, 2021).

Mexico is often described as the pioneer of diaspora engagement in Latin America. The 1991 initiative to repatriate emigrant researchers succeeded considerably with 1,859 researchers having been brought back and retained within 6 years from countries like the United States, Canada, Spain, United Kingdom, and Germany (Tigau, 2018). A network of scientists living abroad was also created in 2002 (Tigau, 2009). Repatriation efforts have also been successful in countries like Singapore, China, and Korea where they could create the infrastructure and network to accommodate the returnees (Meyer and Brown, 1999). Financial subsidies, special schools for kids, preferential treatment in the allocation of positions, and higher salaries were some of the key incentives offered to the returning scientists (Zweig, 2008).

However, not all countries can afford to make investments of this scale; a different approach would be to assume that most of the diaspora would not return and to convert the already existing “sporadic and limited” networks into “multiple, dense and systematic” (Meyer and Brown, 1999). The advantage is that it makes use of the already existing infrastructure and enables availing of the social networks created by the individuals abroad, especially in a professional capacity (Meyer and Brown, 1999); this comes in handy as the willingness to collaborate and trust is advanced by familiarity. China's recent strides in academic research have been attributed to the linkages with its diaspora who increasingly cite papers from China and are cited by domestic researchers (Xie and Freeman, 2020).

The total number of diaspora knowledge networks identified by a 1999 UNESCO paper was around 41 and had been classified into the five categories of “student networks”, “local associations of skilled expatriates”, developing and fully formed intellectual/scientific knowledge networks, and “pooling of expert assistance” through networks like the UNDP-initiated TOKTEN (Meyer and Brown, 1999). TOKTEN stands for Transfer of Knowledge through Expatriate Nationals and allows experts to return to their country of origin for a 2-week to three-month visit where they could assist with the local development issues.

Other well-known networks employed in science diplomacy include the Network of Arab Scientists and Technologists Abroad (ASTA), Argentina's PROCITEXT, Colombian Network of Researchers and Engineers Abroad, Iranian Scholars Scientific Information Network, Irish Research Scientists Association, Latin American Association of Scientists (ALAS), Peruvian Scientific Network, Tunisian Scientific Consortium, and Moroccan Association of Researchers and Scholars Abroad (Meyer and Brown, 1999).

Brazil started by mapping its diaspora and organizing events for celebrating and honoring them; this later grew into addressing specific issues faced by the nation (Maastricht Centre for Citizenship Migration Development, 2021). Such a focused approach is very pertinent from a developing country's perspective. India has seen the involvement of PIOs in projects employing artificial intelligence to solve issues in agriculture and healthcare; coordination on the part of the government would reduce costs and remove the obstacles dissuading diaspora from active engagement currently. This would demand behavioral changes on the part of the diaspora and institutional changes in the country of origin; a connection with the homeland and the genuine desire to solve its developmental issues would have to be inculcated in the minds of the diaspora and bureaucracy back home would have to be streamlined to facilitate diasporic intervention and to create an attractive work environment. A centralized top-down approach would have to be abandoned so that all stakeholders can offer suggestions and a system that is convenient for everyone evolves (Gaillard et al., 2017). This involves multidimensional change and cannot be achieved within a short time.

Paul (2012), while exploring the return of elite expatriate scientists to China, Singapore, Taiwan, and India, identifies funding, administration, network, staff and infrastructure as “elements of the scientific research system” and attitudes toward knowledge, approach to problem-solving, the scope of research ambitions, autonomy, the importance given to seniority and rank, way of communication and approach to differences of opinion as “elements of research culture”, that had to be altered to improve productivity. Governments often focus only on making the research system similar to where the scientists come from, but there is a great scope for mutual learning in a cultural sense as well, demanding an open-mindedness toward new ways of learning and doing things that may organically emerge (Paul, 2012). Something seemingly minor as the reluctance of research scholars to propose a new idea to their supervisors reflects issues with the prevailing academic culture and Indian researchers are shown to have benefited from cross-cultural interactions (Paul, 2012). The ideas, values, and perspectives that the diaspora brings, dubbed “social remittances” by sociologist Levitt (1998), assume significance in this light.

This kind of cultural change can be brought about only in the long term and not merely through programs specifically aimed at science diplomacy. A higher degree of autonomy for research institutions and a freer academic culture help academics become “unintentional diplomats” (Sutton and Lyons, 2014). The private sector, intergovernmental organizations, and civil society actors including alumni networks can step in by facilitating dialogue between multiple scientific communities and creating platforms for collaboration.

The lack of coordination among different actors is a key issue faced by our SD initiatives. Spain has set an excellent example on this front through collaboration between the Ministry of Foreign Affairs and the Ministry of Science and Innovation and between two public agencies CDIT and FECYT (Morena et al., 2017). Scientists with an impeccable understanding of the research landscape in both the host country and Spain are appointed in Spanish embassies to lead the diaspora outreach activities and to foster cooperation between both scientific communities; the already existing diaspora networks had also provided a firm foundation to this initiative (Morena et al., 2017).

Traditional understanding and use of SD stems from state-centric and institutional-centric perspectives. While, there is nothing wrong *per se* with such perspectives, they are not adequate for harnessing the full potential of SD in engaging with Diaspora. A major issue with such perspectives is that they mirror a top to down approach with little scope for a bottom-up perspective. On the other hand, in Climate Change we find that while state-centric science diplomacy is flourishing, there are other developments such as city diplomacy (Bouchet, 2021). In the case of Science Diplomacy also there have been similar developments. According to the S4D4C Project, “New actors become visible in science diplomacy, sub-national regions and cities take charge of addressing global challenges and establish international relationships to exchange experiences and strengthen their profiles” (S4D4C, 2021). Barcelona's Science Diplomacy initiative is a good example of this (Roig et al., 2020).

Cities and sub-national entities may not be as powerful as the states are but they are part of the problem and part of the solution also. For example, while a state may not have a net-zero plan that addresses the climate change issue adequately, a city can draw a road map for a net-zero transition that is quicker and more effective. In the case of STI and engaging with diaspora as we have pointed out, there are initiatives that involve diaspora networks, and formal and informal associations. Initiatives like Global Honduras try to mobilize multiple stakeholders and develop a common agenda and platform for them.

### Initiatives in India

India accounts for one of the largest Diaspora communities, numbering more than 18 million according to the United Nations Department of Economic and Social Affairs (Menozzi, 2021).

In the Indian context, the STIP diaspora has been instrumental in building technology-intensive sectors like information technology and biotechnology. Needless to say, with rapid technological advancements and the advent of the fourth industrial revolution, there is a need to harness the untapped potential of the STI diaspora to realize technological self-reliance and achieve sustainable development goals. India's IT sector has witnessed an exponential boom due to in cross-pollination of ideas and transfer of technological capabilities, wherein the Indian diaspora had been a major driver, in this context.

Efforts are being made by various stakeholders, including the government and private sector to harness the potential of the STI Diaspora in advancing and promoting scientific advancements, at national and international levels. This section reflects on some of the prominent practices to engage with Indian STI Diaspora.

#### Government-Led Initiatives

Developing countries like India have built their STI ecosystem by enhancing their technological capabilities. To promote cross-border technological learning and knowledge sharing, the government has developed various schemes and fellowships to encourage STI Diaspora for participating in national science, research, and development. One such initiative is the *Ramanujan fellowship* (Science and Engineering Research Board SERB, 2019), under the Department of Science & Technology, Government of India. The objective of this fellowship is to engage with Indian scientists and engineers residing in foreign countries and provide them with research positions in India. In 2019-20, 22 fellowships were recommended; some details regarding the fellowship are provided in Table 1.

**Table 1:** Summary of Ramanujan fellowship (2019–2020).

**Broad subject area**	**Number of ongoing awards**	**Number of awards sanctioned during 2019–20**	**Number of project completed during 2019–20**
Chemical sciences	35	2	3
Life sciences	49	4	1
Physical sciences	51	2	2
Mathematical sciences	8	0	0
Engineering sciences	20	0	1
Earth and atmospheric sciences	7	1	1

*Source: SERB, 2019*.

The Department of Biotechnology, Government of India, introduced a similar scheme known as the “Ramalingaswami Re-entry Fellowship” for Indian Nationals, working overseas in biotechnology and life sciences domains who are interested to undertake research positions in India. The fellows are also eligible for regular research grants through extramural and other research schemes of various S&T agencies of the government. In 2021–2022, 88 Ramalingaswami re-entry fellowships were supported by the Department of Biotechnology (Department of Biotechnology, 2021). Various other schemes and fellowships are introduced by the government to engage with STI Diaspora for building scientific prowess at national and sub-national levels. A list is provided in Table 2.

**Table 2:** Fellowships and schemes for Indian researchers residing in foreign countries.

**Srl. No**.	**Name of the fellowship/scheme**	**Description**
1	Visiting Advanced Joint Research (VAJRA) Faculty Scheme	This Scheme is to bring overseas scientists and academicians including Non-resident Indians (NRI) and Overseas Citizen of India (OCI) to India to work in public funded Institutions and Universities for a specific period of time. The scheme offers adjunct / visiting faculty assignments to overseas scientists including Indian researchers to undertake high quality collaborative research in cutting edge areas of science and technology with one or more Indian collaborators.
2	Ramanujan Fellowship	This Fellowship provides attractive avenues and opportunities to Indian researchers of high caliber, who are residing abroad, to work in Indian Institutes/Universities in all areas of Science, Engineering and Medicine. It is directed to scientists and engineers below the age of 40 years, who want to return to India from abroad.
3	Ramalingaswami Re-entry Fellowship	The program is to encourage scientists (Indian Nationals) working outside the country, who would like to return to the home country to pursue their research interests in Life Sciences, Modern Biology, Biotechnology, and other related areas.
4	Biomedical Research Career program (BRCP)	This program provides opportunity to early, intermediate and senior level researchers to establish their research and academic career in Basic biomedical or Clinical and Public Health in India. These fellowships are open to all eligible researchers who wish to relocate or continue to work in India.
	Scientists/Technologists of Indian Origin (STIO) in Indian Research Laboratory	There is a provision to appoint Scientists/Technologists of Indian Origin (STIO) on a contractual basis at Council of Scientific and Industrial Research (CSIR) laboratories to nurture a research field in their area of expertise.
5	Senior Research Associateship (SRA) (Scientist's Pool Scheme)	This scheme is primarily meant to provide temporary placement to highly qualified Indian scientists, engineers, technologists, and medical personnel returning from abroad, who are not holding any employment in India. The Senior Research Associateship is not a regular appointment, but is a temporary facility to enable the Associate to do research/teaching in India while looking for a regular position.
6.	Distinguished and Outstanding scientists scheme for the Scientists and Technologists of Indian Origin	A scheme by CSIR to engage global Indian S and T experts in the organization's activities which focus on shaping a new S and T landscape in India, to address global scientific challenges.
7.	Initiatives of Homi Bhabha National Institute (HBNI)	In this scheme scientists belonging to Indian S and T diaspora may be invited as visiting faculty members to some of the Constituents Institutes (Cis) of Homi Bhabha National Institute. Foreign students can be admitted to any of the five of the constituent institutions of HBNI viz. National Institute of Science Education and Research (NISER). Institute of Physics (loP), Bhubaneswar, Saha Institute of Nuclear Physics (SINP), Kolkata, Harish-Chandra Research Institute (HRI), Allahabad, and Institute of Mathematical Science (IMSc), Chennai.

#### Academic Institutions

To develop indigenous technological capacities, it is important that the sites of academic learning and scientific research promote brain circulation and leverage on capabilities of Indian scientists and researchers residing in foreign countries. To realize this objective, some academic institutions have initiated programs to involve STI Diaspora for capacity building, networking, and mentorship. The alumni association of IIT Madras has envisaged a mentorship program to kick-start the activity. Over 5,000 alumni in 15 countries participated to celebrate the institution's foundation day in 2021 for short lectures on various themes including cutting-edge research and technologies, quantum computing, and extra-terrestrial manufacturing, to name a few (The Hindu, 2021).

Similarly, the IIT Kharagpur Alumni Foundation in the United States has been aggressively promoting student internships of IIT Kharagpur students in foreign institutions for career development as well as to enhance their technological capabilities. It has also led to exposure and learning for Indian students in some world-class academic and research institutions (Basu, 2019).

Some of the institutions are reorienting their recruitment policies to increase the intake of faculty members belonging to the Indian STI Diaspora community. For example, SRM institute has plans to increase the number of NRIs/PIOs among foreign faculty (source: https://www.srmist.edu.in/aboutus/panel-foreign-faculty) (SRM University, 2022).

#### Private Sector

Some private sector companies are also creating avenues to promote linkages with STI Diaspora. Internships are offered by various companies to engage NRI students on projects related to research and development (DNA India, 2016). Consortia like FICCI have been actively involved in events related to the Indian Diaspora, particularly the *Pravasi Bharatiya Diwas*, which are potent avenues to initiate dialogues for collaborations and engagements in STI.

#### Other Interventions

Collaborative efforts of various stakeholders and interventions by non-state actors are also coming into play to collaborate and engage with the Indian STI Diaspora. For example, the Wadhwani AI and WISH foundation are platforms founded by Indian-American technocrats to develop AI solutions for healthcare that are available and accessible for developing countries.

Project Madad, an initiative started by a voluntary group of doctors and professionals from the Indian diaspora in the United States, aims for “proper education and training” of local healthcare workers and registered medical practitioners (RMPs) particularly to manage and respond to the COVID-19 outbreak in rural India (Reuters, 2021). Initiatives like the “India Science Festival” and even Vaibhav Summit open up opportunities to engage and collaborate with Indian scientists and researchers abroad and build linkages with Indian counterparts. These activities have invigorated the dialogue for cross-border technological learning and knowledge sharing (Aggarwal, 2019).

While, there is ample evidence to assert that India is actively encouraging engagements with the STI Diaspora, it is important to note that most of these initiatives are limited in scope and do not have any long-term vision or objectives. Another issue with them is they are not linked with any major initiative in STI or with any technology missions. A fundamental problem with these initiatives is there is no synergy among them and they are envisaged as “stand-alone” programs with a limited objective. It is true that they do help in capacity building but in terms of numbers or scope, these are woefully inadequate for long-term capacity building or institutions acquiring specialized capacity in one field. So, a good question is whether they are adequate to engage with the STEM diaspora or to induce their return to India. We think they are not.

However, for us, the larger problem is these have no linkage with Science Diplomacy and there is no connection between them and various global networks in STI or science academies or with professional associations of scientists and technocrats. As a result, these have become yet another routine activity in terms of fellowships and opportunities than as major attractors for STEM diaspora. Since they are based at and offered by different ministries/institutions/departments without any institution coordinating them or trying to develop a link among them, efforts seem to be fragmented, with each trying to address one need/component. On the other hand, a coherent mechanism that can deal with the needs of STEM diaspora without trying to make them go through these “*Procrustean Bed*s” is what India needs. Given the conditions including age, and disciplines, these function like filtering devices rather than as facilitating doors.

Conspicuous by its absence in all these is Science Diplomacy. Although there is no official Science Diplomacy policy, India has extensive S&T collaborations and is part of big science initiatives. In addition, India is part of groupings like BRICS, QUAD (United States, India, Japan, and Australia) and BASIC. While, there is a strong STI component in the case of the first two, it is not so with BASIC.

Thus, although there are initiatives to engage with the STEM diaspora, these are not adequate and increasing the numbers of fellowships or expanding them is not sufficient, Instead what is needed is a new approach, a new policy, and a strategy.

## Toward a New Model for Development: Linking Science Diplomacy and STI Diaspora

According to Varadarajan, historically the contribution of the Indian diaspora to the development of India's foreign policy has been limited as till the early-1990s, there was no meaningful engagement with the Indian diaspora Varadarajan (2015). So, it is no wonder that India did not have any credible strategy till then to engage with STEM diaspora and benefit from their expertise, etc. While, academics (Pande, 2017) have pointed that out newer forms of engagement with diaspora are in place, there is no coherent and comprehensive approach to engaging with STEM diaspora.

In recent decades, the Government of India has initiated many programs for harnessing the STEM diaspora and for enhancing opportunities for them. But most of them are stand-alone programs and initiatives that serve a narrow purpose. There is no integration or synergy with larger initiatives in STI such as national missions or with sectoral policies that have a strong STI component. While, the absence of an STI policy is a constraint, the larger issue is that of disconnect between SD and programs focusing on NRI/PIOs. There is no official policy on SD. But SD is evident in many instances ranging from Vaccine Diplomacy to the formation of ISA.

The beginning of India's Science Diplomacy can be credited with the APSARA reactor built in 1956 under India-United Kingdom Co-operation Agreement. Since the Science Diplomacy in nuclear technology has seen ups and downs, the 2005 agreement between India-United States on the civil nuclear program is an achievement for Science Diplomacy in India. There are many bilateral research centers based on bilateral cooperation in STIs. At the regional level, there are initiatives like Regional Integrated Multi-Hazard EarlyWarning System (RIMES) for Africa and Asia and The Indian Ocean Global Observing System (IOGOOS) (Goel, 2022). The International Solar Alliance is an international initiative led by India for the effective harnessing of solar energy and the development and adoption of solar energy technologies. India's vaccine diplomacy is a recent phenomenon, and under this, India successfully supplied vaccines to countries all over the world.

The draft STI policy of 2020 states “As for the engagement with Indian diaspora is concerned, the policy direction is to create a fine balance between attracting the best talent back home and creating facilitating channels for the diaspora to contribute to national development from wherever they are appropriate institutional mechanisms and suitable opportunities will be created to engage with the Indian diaspora more effectively” (Department of Science and Technology, 2020). In fact, it states nothing about linking SD with initiatives on NRI/PIOs. In this context, we argue that there is a need to think boldly and imaginatively by going beyond what is being done.

As of now, there is no quality data on STEM diaspora across countries in the world and country-wise data is important as it will help in formulating country-specific strategies. Country-specific strategies are important as they will consider unique features in the respective countries' National Innovation Systems and accommodate the science diaspora accordingly. This will help in developing better bilateral cooperation in STI between India and the other countries. Given India's large number of bilateral cooperation agreements in S&T, India should use Science Diplomacy to enhance their numbers and effectiveness. STEM diaspora can contribute to this and the Government of India can, in turn, with specific policies and programs, try to integrate STEM Diaspora into this.

About two decades ago, in a pioneering study, Khadria (2003) pointed out that there are many networks of STEM diaspora which are part of networks of Indian diaspora and highlighted the contribution of STEM diaspora to S&T in India. There is a need to revisit and study the current status of various networks with which STEM diaspora is associated and whether the current policies and initiatives are adequate to engage with them and tap their potential. In this, the Government of India can work with professional organizations such as Science Academies and various organizations representing scientists in different disciplines. With the involvement of Science Academies, etc., on one hand, and leveraging Science Diplomacy's potential to connect and engage with them on the other hand, better linkages between policies and these professional associations can be developed.

Given the size of India's NRI/PIO and their impact, the Government of India should expand the scope and size of the current ones, to begin with, and go for bigger initiatives that can enable better utilization of their talent and expertise and create a win-win synergy for both. In this, the Central and State Governments can join hands and launch initiatives on themes listed in the Concurrent List of the Constitution of India. For example, in Higher Education, using the expertise and skills of NRI/PIOs can go beyond what is being done now. However, before embarking on this, the following needs to be done:

(1) Consolidate by bringing programs/initiatives with similar/same objectives as a single program.

(2) Establish a single board/authority to deal with all programs in STI related to NRI/PIOs (except those related to admissions to courses).

(3) Examine whether the current programs are adequate in scale and impact vis a vis the STI policy under preparation.

(4) Take a comprehensive view of engaging with STEM Diaspora in all aspects including involving them in incubators and startups.

Regarding employment opportunities for STEM Diaspora, Li et al. (2019) point out that India lacks a comprehensive strategy to fulfill the needs of different forms of returnees nor offers a specific Talent Visa to facilitate their return. According to them, “In India, recruitment plans are skill specific and “pertain to” or “involve” occupations like scientists, technologists and medical personnel. To recruit highly skilled Indians in the diaspora more effectively, we recommend that Indian institutions adopt a more assertive strategy to reach out to talent overseas”.

A more integrated approach, with collaborative efforts from the government and industry would make more effective use of diaspora knowledge networks and facilitate “brain circulation” (Li et al., 2019). Further, their analysis shows that China has programs that have been successful in meeting the objectives of facilitating and enhancing the recruitment of STEM Diaspora (Li et al., 2019). India can examine its policies, and based on a review and adoption/adaptation of successful models from other countries, it can enhance the opportunities to STEM diaspora and ensure that there are all-around benefits for both in the short-, medium-, and long-term. While, Science Diplomacy may not provide an explicit tool for this, the better use of Science Diplomacy in engaging with the STEM Diaspora can result in effective outcomes.

India has committed to SDGs and in achieving them much importance is given to STIs. India is also a pilot country in STI for SDGs Road Mapping. This is a global program involving five countries: Ghana, Ethiopia, Kenya, India, and Serbia. Serbia has better-integrated diasporas with STI policy and has a “Diaspora Cooperation program” under the Science Fund, which is a program for competitive research funding.

The importance of emerging technologies in achieving SDGs is too well-known to be stated here (United Nations, 2021). The success of scientists and technocrats of Indian origin in the ICT industry, AI, and other technologies is well-known. More importantly, the diaspora contributed significantly to the growth of the Information and Communication Technologies sector in India in many ways and ensured that it was a firm footing to meet the global challenges in that sector (Pande, 2014). NRI/PIOs heading mega corporates like Microsoft, Google, and Adobe are too known to be repeated here.

Second generation NRI/PIO scientists are active in many emerging technologies and applications such as bio fabrication (e.g., Ritu Raman of MIT). We have any number of students doing PhD or Post-Doctoral Fellows spread across the world in these fields. The Indian SD is yet to recognize the potential of these emerging leaders and their future contributions. It is because Science Counselors do interact with NRI/PIO scientists and technocrats, and they become yet another constituency/stakeholder in their work. But more is needed in terms of strategy to address the needs of this constituency and identify how their needs can be met while enriching/contributing to the development of emerging technologies in India.

Students, particularly doctoral and post-doctoral fellows can be considered stakeholders in Science Diplomacy and engaging with them and early career scientists can help in their involvement in Science Diplomacy and contribute to that. There are some good examples of this such as EURAXESS North America (Science Policy Exchange, 2018). Given the large number of students and post-doctoral fellows from India, making them stakeholders in Science Diplomacy is a good idea.

India should appoint Science Ambassadors and Science Evangelists who will go beyond what a typical science counselor would do. These Ambassadors and Evangelists can be chosen from scientists/technocrats or entrepreneurs. They will act as a bridge between the Government of India and the NRI/PIO diaspora and will focus on a particular region. It is also possible that the Ambassadors or Evangelists can work on broad disciplines and connect the NRI/PIOs with programs and initiatives. We illustrate this with an example. India has many programs and initiatives on AI and there is a national plan for AI. There are also state-level initiatives on AI and other stakeholders such as industry, NASSCOM is working with state and central governments' initiatives. But if one looks at the existing programs or initiatives there is nothing specific that will engage with NRI/PIOs in this. This is because while there are programs and initiatives on NRI/PIOs, they are almost general/generic in nature without any specific orientation toward any specific technology or Technology Mission.

Simultaneously, the Technology Missions or related initiatives are specific in terms of objectives and goals but have no specific plan or role assigned to NRI/PIOs. A Science Ambassador or Evangelist can address this gap effectively by acting as a bridge among these. For example, in the case of the use of AI in school education, the Ambassador in San Francisco will map the available expertise among NRI/PIOs in the West Coast region and identify the scope for their engagement with relevant programs and initiatives. (S)he will develop a forum for continuous engagement of NRI/PIOs with relevant stakeholders and ensure that their expertise is understood and used. It is not necessary that an NRI/PIO should be fully engaged with such programs or initiatives. Given their expertise in that domain, they can guide the stakeholders in taking the right decisions including deploying the appropriate technology.

On the other hand, mapping the expertise will also help in identifying opportunities for startups and not for profit initiatives to contribute to such initiatives /missions. For example, a Science Ambassador focused on AI can work with the central government and state government in this. (S)he can enable them to get connected to relevant experts and can act as a bridge and catalyst. Although there are initiatives like developing databases on experts, and, these can be used by Technology Missions or state governments, databases or using them is not sufficient. What is needed is personal interaction and engagement with NRI/PIO experts and continuous attention to the developments.

Databases are often static and do not get updated nor can capture developments like an NRI/PIO getting a patent on a technology that is relevant for a mission. Updating them on a regular basis is one solution but that alone will not help. A Science Ambassador can bring in a personal touch and combine that with expertise and goodwill to make a real difference. (S)he can facilitate processes to move faster or enable quicker interaction.

But given the large number of NRI/PIOs on one hand and the limited resources available with Embassies and Consulates on the other, there will be real constraints. So, a Science Ambassador/Evangelist must be part of a larger strategy of engaging with NRI/PIOs and STI. That larger strategy should be embedded in the overall strategy on NRI/PIOs. It is here that SD can play a positive and effective role.

The traditional view of “Science for Diplomacy” and “Diplomacy for Science” is necessary but not sufficient because both Diplomacy and Science are not broadly construed in them. Concepts like Diaspora Diplomacy are useful and they have to be rethought in terms of Science Diplomacy and STI for Development. Similar to “Science for Diplomacy” and “Diplomacy for Science”, there is “Diplomacy by Diasporas” and “Diplomacy through Diasporas” (Ho and McConnell, 2017). Ho and McConnell (2017) point out that “the transnational and scalar actions of diasporas point to the multi-directional aspects of diaspora diplomacy. This leads us to conceptualize diaspora diplomacy as diaspora assemblages composed of states, non-state and other international actors that function as constituent components of assemblages, connected through networks and flows of people, information and resources” (Ho and McConnell, 2017).

In recent years, diaspora diplomacy has attracted the attention of policymakers and academics and the literature on this is exploring inter alia, how diaspora diplomacy and public diplomacy can be linked and used (Rana, 2013). However, there is not enough literature on using diaspora diplomacy and Science Diplomacy together. The fluid nature of diaspora diplomacy can result in contentious outcomes and as often who constitutes the real and authentic diaspora and who cannot be considered so can be controversial. But in the case of linking Diaspora Diplomacy with Science Diplomacy, such a situation will not arise as the objective here is different and is focused on expertise, knowledge, and their usefulness than on identity politics or claims over identity. In issues like Climate Change, there are sub-national actors who have emerged and are addressing an issue of common concern by developing networks, sharing information, and building alliances that transcend national borders.

Diasporas can be compared with Janus. Just as in the case of Janus, they look in two directions, one is the direction of their origin and another is that of their location or place of flourishing. But they can remain “at home” in both places and link their country of origin and their country of work/doing business. According to Burns (2013):

“In the United States, a quarter of foreign-born workers with college degrees work as scientists or engineers. According to a report by the Kauffman Foundation, foreign-born entrepreneurs started a quarter of U.S. technology startups over the past six years. In Silicon Valley alone, 44 percent of these engineering and technology ventures were founded by at least one immigrant. As of 2010, one-third of the 314 laureates who won their Nobel Prizes while working in the United States were foreign-born.”

While, this shows the importance of diasporas, organized diasporas with their collective expertise, networks, and linkages can play a key role in linking diaspora diplomacy and Science Diplomacy in both countries. For this, we need to think beyond state-centric and science institutions-centric Science Diplomacy and think in terms of new constellations, collectives, and assemblages. For example, Diaspora Diplomacy combined with Science Diplomacy can result in developing a mechanism for connecting STEM diasporas based in Silicon Valley with a particular state that wants to utilize their expertise in solar energy or renewable energy and integrate that with its energy and climate change plan. In this case, only a section of the STEM diasporas will be involved with that state. But such an engagement can be part of the state-level Science Diplomacy strategy which may or may not have linkages with the national-level one. What is true of solar energy or renewable energy can be true of AI.

At another level, this can be linked with state-level SDGs also in this case SDG 7, i.e., affordable and clean energy. The engagement can have different components including capacity building, investment, venture capital, transfer of technology, and supporting startups.

The approach we propose envisages the following:

- Broadening of Science Diplomacy in terms of approach and strategy.
- Involvement of stakeholders including states.
- Linking Diaspora Diplomacy with Science Diplomacy with a focus on STI, SDGs, technology Missions, and other goals/objectives.
- Creating new institutional structures, networks, and assemblages.
- Specific engagement with STEM diaspora including their networks, associations, and resources.
- Developing a coherent policy and strategy that creates an enabling environment for effective use of and engagement of STEM diaspora.

Science Ambassadors or Evangelists will be part of this proposed structure. But in the absence of a supportive environment and institutional arrangements, they cannot do much. The supporting environment has to be created through the strategy while institutional arrangements have to be made. These can be built upon the available ones or can be new initiatives. For example, in addition to traditional professional associations and networks, there can be Associations of NRI/PIO like professional associations. An association of NRI/PIOs engaged in solar energy can bring together stakeholders of different kinds including entrepreneurs and scientists. The association can support the work of Science Ambassador or Evangelist on one hand, and, on the other hand, can engage with the state that is keen to harness their expertise, knowledge, and networks. The Science Ambassador can act as a connecting point or bridge among the other stakeholders. (S)he can link up with International Solar Alliance (ISA) and similar organizations. Given the increasing importance of renewables in decarbonization strategies and various bilateral and multi-lateral initiatives in this, using Science Diplomacy to build bridges and alliances can make a difference in translating plans into actions and achievable targets. India, for example, can have a Science Ambassador for Solar Energy, positioned strategically in countries like United States and China to facilitate developing alliances and networks. We are aware that this needs a detailed plan and that is beyond the scope of this article.

However, the problem here is that, although there are many diaspora associations based on different identities and for different purposes, there is hardly anything that is related to STI and development with the objective to work with different stakeholders in India and abroad through Science Diplomacy. Hence there is a need to relook at the current associations and networks of the Indian diaspora including the professional networks and associations. In the absence of any functional association or network, the Government of India, and other stakeholders can initiate the process of forming them. For example, an association of NRI/PIOs working on quantum technologies or an association of NRI/PIOs working on AI. In both cases, there are national-level missions/plans and state-level ones. But to have a focus on themes the association(s) can have sub-groups or working groups that can be linked with goals or specific programs. At another level, these associations can form a Federation of (such) Associations that will be a coordinating body that can play a larger role in the larger context of STI.

If India's Science Diplomacy policy and strategy must harness on STEM diaspora, it has to think in terms of new institutions, networks, and objectives. It needs a Mission-oriented approach. The role of the current programs and initiatives can be rethought considering the above discussion. For example, the development of databases of NRI/PIOs can be linked to that of forming associations and use of Diaspora Diplomacy by making NRI/PIOs key stakeholders in this, rather than as passive information sharers. They should have a role and say in developing and using them. Similarly, mapping of contributions of NRI/PIOs in terms of emerging technologies should be undertaken and this should include patents. When the contribution of NRI/PIOs in a particular field is mapped in terms of technologies and applications, it can be compared with technologies needed or expertise in demand in India or with the state of the art in India. Such an analysis will reveal the potential of NRI/PIOs to contribute collectively or individually to ongoing research or projects in India. In another way, this can relate to research problems or issues in adoption or commercialization. These exercises need much technical expertise and perhaps also involve inter-disciplinary research. They also need institutional structures and programs to do them continuously.

One major shift that is needed is that instead of relying on top-down Science Diplomacy, India should make effective use of bottom-up approaches in Science Diplomacy. In this regard, Spain's initiatives in giving support to bottom-up associations and the adoption of a multi-stakeholder approach have been effective (Morena et al., 2017). India can learn from such examples and develop a strategy that is commensurate with its objectives and resources.

In this article, we have outlined some of the possibilities and an approach that can be useful for Indian Science Diplomacy. Certainly, these need to be developed further and sharpened and our idea is to highlight the potential and possibilities. Developing them further is reserved for another occasion/opportunity.

## Conclusion

Our analysis shows that although many initiatives have been taken by the Government of India to make the best use of expertise, knowledge, and connections of the STEM Diaspora, there is hardly any synergy or coherence among them. Nor are they linked with major initiatives or Missions or for that matter with SDGs. One reason perhaps is India has no specific and explicit policy on Science Diplomacy. Another could be that different ministries and departments are managing these initiatives with no central mechanism or agency to coordinate them. Not all initiatives related to diaspora need to have an STI component in them or be targeted toward STEM diaspora. But what is crucially missing is the policy and strategy on using Science Diplomacy for sustained and sustainable engagement with them. Some initiatives like the creation of databases and having dialogues with the STEM diaspora are important but the possibility of them getting stagnant or a routine affair cannot be ruled out. Science Counselors and attaches have an important role in engaging with STEM Diaspora but we have suggested that India needs “Science Ambassadors” and “Evangelists” for the specific purpose of engaging with STEM diaspora.

Based on the literature and examples from other countries and in other contexts, we have called for a new approach to using Science Diplomacy, going beyond the traditional state-centric Science Diplomacy and suggesting better linkages between diaspora diplomacy and Science Diplomacy. Science Diplomacy itself is changing and there is a strong case for “Data Driven” “Science Diplomacy 2.0” (Turchetti and Lalli, 2020). Our approach is that Science Diplomacy should use strategically the data regarding STEM diaspora going beyond the creation of and usage of NRI/PIO databases.

For effective harnessing of expertise, knowledge, and networks of STEM diaspora, India's Science Diplomacy policy and strategy should adopt a Mission mode approach and think in terms of developing relevant and new institutions and networks. Some aspects of this have been discussed and elaborated upon in this article. It is hoped that this will kindle new thinking on this subject among the experts and policymakers.

## Author Contributions

The article has been conceptualized and planned by NP and KS. TD was involved in providing inputs to the arguments and organizing the article. All authors contributed to the article and approved the submitted version.

## Conflict of Interest

The authors declare that the research was conducted in the absence of any commercial or financial relationships that could be construed as a potential conflict of interest.

## Publisher's Note

All claims expressed in this article are solely those of the authors and do not necessarily represent those of their affiliated organizations, or those of the publisher, the editors and the reviewers. Any product that may be evaluated in this article, or claim that may be made by its manufacturer, is not guaranteed or endorsed by the publisher.
